# Transcriptomic Profiling Reveals a Role for TREM-1 Activation in Enterovirus D68 Infection-Induced Proinflammatory Responses

**DOI:** 10.3389/fimmu.2021.749618

**Published:** 2021-11-23

**Authors:** Jinyu Li, Shan Yang, Sihua Liu, Yulu Chen, Hongyun Liu, Yazhi Su, Ruicun Liu, Yujun Cui, Yajun Song, Yue Teng, Tao Wang

**Affiliations:** ^1^ School of Life Sciences, Tianjin University, Tianjin, China; ^2^ State Key Laboratory of Pathogen and Biosecurity, Beijing Institute of Microbiology and Epidemiology, Beijing, China; ^3^ Institute of Tianjin Key Laboratory of Function and Application of Biological Macromolecular Structures, Tianjin, China

**Keywords:** Enterovirus D68, transcriptome, TREM-1 signaling, proinflammatory response, NF-κB signaling

## Abstract

Increasing cases related to the pathogenicity of Enterovirus D68 (EV-D68) have made it a growing worldwide public health concern, especially due to increased severe respiratory illness and acute flaccid myelitis (AFM) in children. There are currently no vaccines or medicines to prevent or treat EV-D68 infections. Herein, we performed genome-wide transcriptional profiling of EV-D68-infected human rhabdomyosarcoma (RD) cells to investigate host-pathogen interplay. RNA sequencing and subsequent experiments revealed that EV-D68 infection induced a profound transcriptional dysregulation of host genes, causing significantly elevated inflammatory responses and altered antiviral immune responses. In particular, triggering receptor expressed on myeloid cells 1 (TREM-1) is involved in highly activated TREM-1 signaling processes, acting as an important mediator in EV-D68 infection, and it is related to upregulation of interleukin 8 (IL-8), IL-6, IL-12p70, IL-1β, and tumor necrosis factor alpha (TNF-α). Further results demonstrated that NF-κB p65 was essential for EV-D68-induced TREM-1 upregulation. Moreover, inhibition of the TREM1 signaling pathway by the specific inhibitor LP17 dampened activation of the p38 mitogen-activated protein kinase (MAPK) signaling cascade, suggesting that TREM-1 mainly transmits activation signals to phosphorylate p38 MAPK. Interestingly, treatment with LP17 to inhibit TREM-1 inhibited viral replication and infection. These findings imply the pathogenic mechanisms of EV-D68 and provide critical insight into therapeutic intervention in enterovirus diseases.

## Introduction

Enterovirus D68 (EV-D68), an emerging pathogen in humans, is a member of the *Picornaviridae* family (1, 2), and causes a wide range of respiratory symptoms and a series of severe complications. In 2014, hundreds of children in 46 states of the USA and the District of Columbia came down with a respiratory illness caused by EV-D68, but there had been few reports of EV-D68 infection prior to this. Notably, previous studies has concluded that EV-D68 has become the predominant enterovirus type in hospitalized children with respiratory symptoms in Europe (3, 4) and Japan (5, 6). Although EV-D68 has the potential to become a serious public health threat to children, the vaccines or medicines to prevent or treat EV-D68 infections is still not available.

Multiple EV-D68 outbreak and pathogenicity reports highlight the need to understand the molecular mechanisms to develop countermeasures against virus infection. The innate immune system is critical to the host’s resistance to viral infection. When challenged by pathogens, host cell pattern recognition receptors (PRRs) can recognize viral components through the pathogen-associated molecular patterns of viruses or other pathogens, and subsequently trigger intracellular signaling cascades to activate proinflammatory responses, which can induce an antiviral state in infected host cells (7, 8). For evading and antagonizing the host innate immune response, viruses have evolved complex mechanisms (6). Previous studies have reported that enterovirus has several inhibition mechanisms to diminish production of type I interferon, resulting in reduced host antiviral responses (9–11). As a feature of their pathogenic mechanism, many viruses facilitate their replication by interacting with host factors. Our recent studies found that stress granule proteins interact with the 3’-untranslated region of EV-D68 to block viral replication (12). Furthermore, our results indicated that EV-D68 2A^pro^ cleaves TRAF3, inhibits type I interferon responses, and subverts the innate immune responses (13).

The host-EV-D68 interplay signaling pathways are highly complex, in which the genome-wide transcriptional profiling (RNA-Seq) would increase understanding of the molecular mechanism. In this study, we applied RNA-seq to human muscle rhabdomyosarcoma (RD) cells infected with EV-D68 over multiple timepoints. The results showed that the highly activated TREM-1 was involved in inflammatory cytokines production during EV-D68 infection. And the activation of TREM-1 associated with NF-κB p65 was identified by siRNA treatments and a dual-luciferase assay. Finally, based on large-scale proteomic screen arrays, we demonstrated that TREM-1 mainly transmitted activation signals to phosphorylate p38 MAPK to influence inflammatory responses. These findings contribute to the understanding of pathogenic mechanisms of EV-D68, and help to develop novel therapeutic strategies in enterovirus diseases.

## Materials and Methods

### Cells and Virus

Rhabdomyosarcoma (RD), HEK293T, and Hela cells were cultured in Dulbecco’s modified Eagle’s medium (DMEM) supplemented with 10% fetal bovine serum and 1% penicillin-streptomycin (cat. no SV30010, Hyclone, Logan, UT, USA) at 37°C in 5% CO_2_ atmosphere. The EV-D68 Fermon strain (GenBank accession no. KU844179.1) was a gift from Xiaofang Yu (Johns Hopkins University, Baltimore, MD).

### Reagents and Antibodies

TREM-1 (NM_018643) was cloned into the pcDNA3.1 vector to incorporate a 3x FLAG-tag on the C-terminus. The plasmid was confirmed by DNA sequencing analysis. Synthetic peptide LP17 (LQVTDSGLYRCVIYHPP, GUOTAI Bio, Beijing, China) was used to treat RD cells at a final concentration of 1 μg/ml or 1.5 μg/ml. The same dilution of dimethylsulfoxide (DMSO, cat. no D8418, Sigma-Aldrich, Saint Louis, USA) was used as a negative control. A Cell Counting Kit 8 (CCK8) was purchased from Solarbio (cat. no CK04, Beijing, China). Rabbit polyclonal anti-TREM-1 antibody was purchased from Abcam (cat. no ab104413, Abcam, Cambridge, MA). Rabbit polyclonal antibody recognizing NF-κB P65 (cat no. 10745-1-AP) and Flag-tag Polyclonal Antibody (cat no. 20543-1-AP) were purchased from Proteintech (ProteinTech Group, Chicago, IL, USA). β-Tubulin mouse monoclonal antibody was purchased from Sungene Biotech (cat no. KM9003T, Tianjin, China). Pyrrolidine dithiocarbamate (PDTC) was purchased from MCE (cat no. HY-18738, MedChemExpress, Monmouth Junction, NJ).

### Tissue Culture Infective Dose 50 (TCID50) Assay

The median tissue culture infective dose (TCID50) assay was used to determine the multiplicity of infection (MOI) of the virus in RD cells. For cell infection, when RD cells grew to 80% in a 10 cm dish, EV-D68 was added at an MOI of 0.1. The cytopathic effect (CPE) caused by EV-D68 infection was observed by light microscopy at different hours post infection (hpi) using an Olympus CKX31 microscope (Olympus). Working stocks (1*10^6.25^ PFU per ml) were stored at 80°C.

### RNA-Seq Data Analysis

RD cells (~80% confluence) infected with or without EV-D68 Fermon for 6 h, 12 h, or 24 h were harvested and lysed using TRIzol solution. At least three replicates were included for each experimental group. Genome-wide RD cell differential gene expression profiling was performed using RNA-seq by Novogene Co. Ltd. (Tianjin, China). Sample sequencing libraries were generated using an NEBNext Ultra RNA Library Prep Kit for Illumina, and Sequenced by synthesis on a *HiSeq* 2500 instrument (Illumina, San Diego, CA, USA) according to the manufacturer’s instructions. To ensure the quality and reliability of data analysis, it was necessary to filter the original data, including removing reads with adapters and low-quality. HISAT2 software was used to map compare clean reads with the reference genome. Next, we obtained original read count of all genes in each sample, and calculated the corrected expression values Fragments Per Kilobase per Million (FPKM) based on sequencing depth and gene length. We analyzed the correlation between different groups using principal component analysis (PCA) and Pearson correlation analysis to determine the authenticity of the data.

The threshold values |log2 (Fold Change)| >1 & adjusted p-value (padj) <0.05 were used to identify differentially expressed genes (DEGs) relative to the mock group. Finally, we performed statistical and significance analyses on DEGs in different comparison groups. Cytoscape software v3.2.1 (cytoscape.org) was used to integrate target genes-transcriptional factors regulatory network, an open-source software package for visualizing complex networks and integrating these networks with any type of attribute data.

### Total RNA Extraction and Real-Time PCR

Total RNA from cells was extracted using Qiazol (cat. no 79306, Qiagen) according to the manufacturer’s instructions, and concentrations were quantified with a Nanodrop 1000 spectrophotometer (Thermo Scientific). Total RNA was reverse-transcribed into cDNA with TransScript First-strand cDNA Synthesis SuperMix (Cat. no AT301, Transgene). Next, cDNA was subjected to Quantitative Real-time PCR (RT-PCR) to measure mRNA levels using a TransStart Top Green qPCR SuperMix Kit (Cat. no AQ131, Transgene) according to the manufacturer’s protocol. Relative mRNA expression levels were normalized against glyceraldehyde-3-phosphate dehydrogenase (GAPDH). Cycle threshold (Ct) values were determined for TREM1, IL-1β, IL-6, IL-8, IL-12 p70, TNF-α, TNF-κB p65, and GAPDH. The fold change relative to control samples for each gene was determined using the 2^-ΔΔCt^ method. The primers for the above genes are listed in **Supplementary Table 1**.

### Ingenuity Pathway Analysis (IPA)

IPA is a novel causal analysis approach based on a large-scale causal network derived from the Ingenuity Knowledge Base, including ‘Upstream Regulator Analysis’, ‘Mechanistic Networks’, ‘Causal Network Analysis’ and ‘Downstream Effects Analysis’ function modules (14–17). IPA can integrate published upstream regulators and downstream targets of specific molecules. IPA use Z-score as both a significance measure and a prediction of the activation state of the specific regulator. We performed core analysis and pathway generation based on mRNA fold changes from RNA-seq experiments using IPA software (Qiagen, Redwood City, CA).

### Western Blotting

Cells were harvested and lysed with radioimmunoprecipitation assay (RIPA) lysis buffer. After heating at 99°C for 10 min to denature the protein, SDS-PAGE was performed to separate proteins, and a semidry transfer membrane was employed to transfer proteins onto a nitrocellulose membrane. The membrane was blocked with 5% skim milk for 1 h at 37°C, and incubated at 4°C for 12 h with primary antibody. The next day, the membrane was incubated with the corresponding secondary antibody at room temperature for 1 h, and finally scanned using a Fluorescence & Chemiluminescence Gel Imaging System (Peiqing, Shanghai, China). Protein bands were quantified using ImageJ software (version 1.53, National Institutes of Health, Bethesda, MD, USA).

### Milliplex Analysis

Inflammatory cytokine levels of uninfected and infected RD cells were measured using a MILLIPLEX assay kit (Cat. no HCYTA-60K, Millipore, Sigma) and a MAGPIX Multiplexing System (Millipore, Sigma) following the manufacturer’s instructions. Data were analyzed using xPONENT4.2 and Milliplex Analyst 5.1 data analysis software (Millipore, Sigma).

### Immunofluorescence and TUNEL Assay

The effect of PDTC on EV-D68-infected RD/Hela cells was investigated by immunofluorescence assay (IFA). Cells were incubated with 4% paraformaldehyde for 15 min and permeabilized with 0.5% Triton X-100 for 10 min. They were then washed in phosphate-buffered saline (PBS) and blocked with 1% bovine serum albumin for 20 min. Samples were then incubated with the corresponding primary antibody for 1 h at 37°C or overnight at 4°C, followed by secondary antibody at 37°C for 40 min. Finally, nuclei were stained with 4’,6-diamidino-2-phenylindole (DAPI) for 10 min, and immunofluorescence was observed under a confocal microscope.

Apoptosis was measured using the terminal deoxynucleotide transferase dUTP nick end labelling (TUNEL) assay with an APO-BrdU TUNEL Assay Kit (Molecular Probes, Eugene, OR, USA) through labelling of the 3’ end of fragmented DNA isolated from apoptotic cells. TUNEL assays were performed according to the manufacturer’s instructions. All slides were observed under a fluorescence microscope. Stained cells were examined under an Olympus confocal microscope (Olympus).

### Dual-Luciferase Assays

The 2000 bp region before the TSS site was selected as the promoter region of TREM-1 and cloned into the pGL3-Basic vector to generate a firefly luciferase reporter plasmid Pro_TREM-1. Next, NF-κB p65 or pcDNA3.1 was cotransfected with Pro_TREM-1 or pGL3-Basic in HEK293T cells (24-well plate). All experimental groups were transfected with pRL-sv40. After 48 h, luciferase activity in cell lysates was determined by dual-luciferase reporter assay. Renilla luciferase activity was used as a control for firefly luciferase activity.

### Phospho-Specific Protein Microarray Analysis

Phospho-array detection was performed in collaboration with Wayen Biotechnology (Shanghai, China). Proteins were extracted from RD cells, labeled with biotin, and hybridized to the Phosphorylation ProArray (Full Moon BioSystems, Sunnyvale, CA, USA) using an Antibody Array Kit (Full Moon BioSystems) to determine the specific signaling phospho-antibody profiles. Finally, we scanned the corresponding fluorescence intensity using a GenePix 4000B instrument (Axon Instruments, Houston, TX, USA) using GenePix Pro 6.0 software to acquire phosphorylation levels for specific proteins. In our study, Ratio ≥1.15 was the threshold for upregulation and Ratio ≤1/1.15 was the threshold for downregulation.

### Small Interfering RNA Knockdown

The si-p65 sequence was obtained from previous research (18) and synthesized by Sangon Biotech (Shanghai, China). Cells (24-well plates, ~80% confluence) were transfected with si-p65 and nonspecific siRNA (100 nM) using Lipofectamine 2000 (cat. no 11668019, Invitrogen, Carlsbad, CA). The knockdown efficiency of si-p65 was examined by western blotting (WB) and RT-PCR.

### Statistical Analysis

All data were from three independent experiments and were shown as the mean ± standard deviation (SD). The statistical significance of differences among mean values of multiple groups was analyzed by a one-way analysis of variance (ANOVA) using Statistical Package for the Social Sciences (SPSS) version 25 (Chicago, IL, USA). All data were visualized using GraphPad Prism (GraphPad Software, version 6.01, San Diego, CA, USA). *p <0.05, **p <0.01, ***p <0.001.

## Results

### Transcriptome Landscape Analysis Reveals Functional Regulation by Hosts in Response to EV-D68 Infection

**Figure 1:**
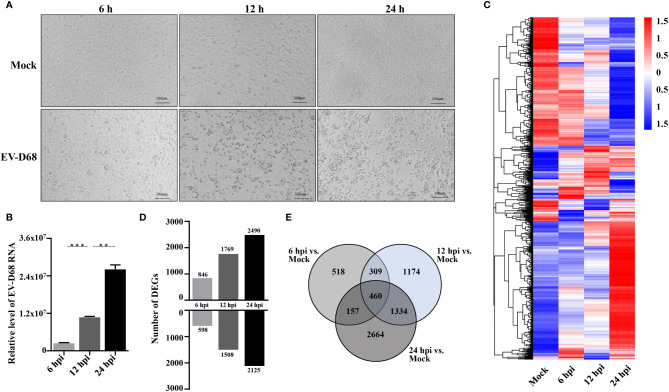
Transcriptome landscape revealing host functional regulation in response to EV-D68 infection. **(A)** Detection of EV-D68-induced CPE. RD cells were infected with EV-D68 (MOI = 0.1), and were imaged *via* light microscopy at 6 h, 12h, and 24 h post-infection. **(B)** Replication of EV-D68 in RD cells. Cells in 24-well plates were infected with EV-D68 (MOI = 0.1) and collected at designated time points (6 hpi, 12 hpi, and 24 hpi). RT-PCR measurement of relative viral RNA levels. **(C)** Clustering heatmap showing differential gene expression between mock and EV-D68-infected groups. The ordinate is the normalized FPKM expression values of differential genes. Different colors show gene expression level differences, with high expression colored red and low expression colored blue. The closer the color distributions of the two samples, the more similar the expression patterns. **(D)** The number of DEGs at different infection time points. **(E)** Venn diagrams showing overlapping DEGs between different comparison groups. **p <0.01, ***p <0.001.

We aimed to determine global gene expression profiles to investigate how EV-D68 shapes the host transcriptional response. The EV-D68-susceptible RD cell line was used as an infection model of EV-D68, in which EV-D68 is capable of normal replication and proliferation (19, 20). We collected infected cells at different time points (6 h, 12 h, and 24 h), hoping to observe the dynamic mutual regulation between EV-D68 and infected cells (multiplicity of infection [MOI] = 0.1). In the early stages of virus infection, the cytopathic effect (CPE) was relatively insignificant. At 12 h post-infection (hpi), RD cells began to shrink, and some were shed (**Figure 1A**). At 24 hpi, EV-D68 infection promoted extensive cell rounding and detachment of RD cells, consistent with the CPE caused by enterovirus infection (1, 19). **Figure 1B** RT-PCR result showed the replication of EV-D68 in RD cells. Next, total RNA from non-infected and EV-D68-infected RD cells (6, 12, and 24 hpi) was collected and analyzed using RNA sequencing (RNA-seq) to quantitate host gene expression. From an overall perspective, a clustering heatmap was drawn to show gene expression levels at the indicated timepoints upon EV-D68 infection (**Figure 1C** and **Supplementary Data Sheet  1**). Differential expression is shown as color depth, with high expression colored red and low expression colored blue, respectively. The threshold values |log2(Fold Change)| >1 & padj <0.05 were used to identify DEGs relative to the mock control group. We identified 6,616 DEGs, including 1,444, 3,277, and 4,615 at 6 hpi, 12 hpi, and 24 hpi, respectively. Detailed DEGs list of all comparison group can be found in **Supplementary Data Sheet 2** (6 hpi vs. Mock), **Supplementary Data Sheet 3** (12 hpi vs. Mock), **Supplementary Data Sheet 4** (24 hpi vs. Mock). Differential expression analysis for three comparison groups, mock vs. 6 hpi, mock vs. 12 hpi, and mock vs. 24 hpi, identified 1,508, 1,863, and 1035 DEGs, including 598, 15,08, and 2,125 upregulated genes and 846, 1,769, and 2,490 downregulated genes, respectively (**Figure 1D**). We also classified overlapping or unique DEGs to illustrate the differences between different timepoints post-EV-D68 infection, as summarized by Venn diagrams (**Figure 1E**).

To comprehensively understand the gene function changes upon EV-D68 infection, Gene Ontology (GO) analysis was carried out on DEGs upregulated at 6 hpi, 12 hpi and 24 hpi, and annotations searched using the GO database. GO enrichment analysis identified related biological process (BP), cellular component (CC), and molecular function (MF) categories. Upregulated DEGs at 6 hpi were primarily associated with GO terms related to the cellular response to external stimulus, NF-κB signaling, negative regulation of kinase activity, and regulation of transcription regulatory region DNA binding (**Supplementary Figure 1A**). The majority of upregulated DEGs at 12 hpi were associated with biological processes including apoptotic signaling pathway, NF-κB signaling, response to endoplasmic reticulum stress, regulation of DNA binding transcription factor activity, cellular components (focal adhesion, cell-substrate junction, cytoskeleton, transcription factor complex), and molecular functions such as DNA binding activity, transcription activator, and corepressor activity (**Supplementary Figure 1B**). Most upregulated DEGs at 24 hpi were assigned to BP subcategories viral gene transcription and expression, and protein synthesis and processing, CC subcategories cytosolic ribosome and DNA packaging complex, and MF subcategories DNA binding activity, transcription activator and corepressor activity, similar to the GO enrichment results at 12 hpi (**Supplementary Figure 1C**).

### Activated TREM-1 Signaling Is Involved in Inflammatory Responses During EV-D68 Infection

**Figure 2:**
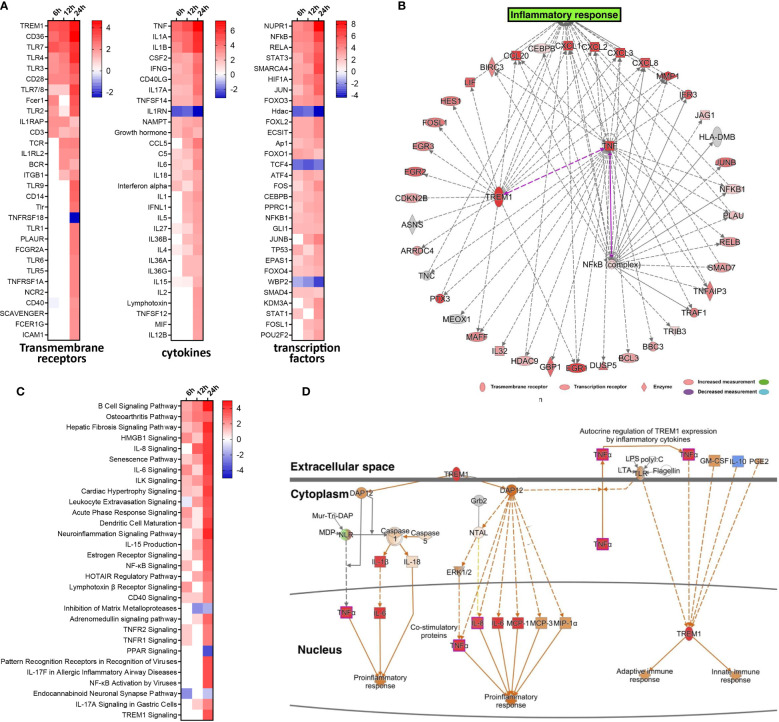
Activated TREM-1 signaling is involved in inflammatory responses during EV-D68 infection. **(A)** The top 30 upstream regulators identified based on z-score values, including transcription factors, transmembrane receptors and cytokines. Different colors represent the corresponding regulator Z-scores, with activated colored red and inhibited colored blue. **(B)** GRN at 24 hpi. Target genes of each regulator were used to perform a GRN analysis. Red or pink = upregulated, gray or white = does not meet cut-off criteria or not involved in the pathway. Solid arrows = direct interaction, dotted arrows = indirect interaction. **(C)** The top 30 enriched signal pathways identified based on activation z-scores at indicated timepoints. Different colors represent the corresponding signal pathway Z-scores, with activated colored red and inhibited colored blue. **(D)** The activation level of TREM-1 and downstream inflammatory cytokines at 24 hpi were determined by activation z-scores. TREM-1 pathway analysis by IPA. Red or pink = upregulated, green = downregulated), gray or white = does not meet cut-off criteria or not involved in the pathway. Solid arrows = direct interaction, dotted arrows = indirect interaction.

To gain insight into the underlying gene regulatory networks against EV-D68 infection, we performed activation analysis of DEGs based on the z-scores. First, three comparisons were made at indicated timepoints of EV-D68 infection, including transmembrane receptors, transcription factors, and cytokines (**Figure 2A**). We found that several immune-related genes, including TLR family members (TLR7, TLR4, TLR3), TNF family members, IL-1 family members, and IFN and some transcription factors (NF-κB, STAT3, JUNB) were significantly differentially regulated. Among these, transcription factor nuclear factor kappa B (NF-κB), the transmembrane receptor TREM-1, and the cytokine tumor necrosis factor alpha (TNF-α) showed increased activation from 6 to 24 hpi. Next, the three regulators and their target genes were used to reconstruct a gene regulatory network (GRN) in **Figure 2B**. The GRN results confirmed a positive feedback loop among the three genes, which is likely to cause significant activation of TREM-1 at 24 hpi. Furthermore, the dynamic GRN for the 24 hpi also demonstrated that activated TREM-1 promotes a severe inflammatory response in which highly activated upstream and downstream regulators are related to inflammation. Thus, the upstream regulators involved in viral infection are likely to utilize the TREM-1 signaling pathway to mediate inflammatory responses in EV-D68 infection.

We subsequently performed pathway enrichment analysis to explore the possible mechanistic functional pathway during EV-D68 infection. The top 30 enriched pathways are listed according to activation z-score values in **Figure 2C**. Additionally, we found that diverse antiviral immune responses were activated, including PRRs that recognize viruses, HMGB1 signaling pathway, IL-8 signaling pathway, IL-6 signaling pathway, TNFR signaling, acute phase response signaling, and others (**Figure 2C**). Consistent with the results of upstream regulator analysis, NF-**κ**B signaling, and TNFR signal pathway were activated at 6 hpi, and persisted until 24 hpi. Remarkably, TREM-1 signaling was most strongly activated at 24 hpi. Therefore, dynamic TREM-1 signaling was observed at 24 hpi (**Figure 2D**). TREM-1 directly interacts with DAP12 to trigger distinct downstream signal transduction pathways for proinflammatory responses (21). The expression of genes related to these pathways, such as TNF-α, IL-6 and IL-8, was altered during viral infection. This implies that the TREM-1 pathway may be involved in the inflammatory cytokine response at 24 hpi during EV-D68 infection. Of note, the molecular mechanisms related to the TREM-1 pathway have not been investigated previously in the context of proinflammatory responses during EV-D68 infection.

### TREM1 and Its Downstream Cytokines Are Upregulated in Response to EV-D68 Infection

**Figure 3:**
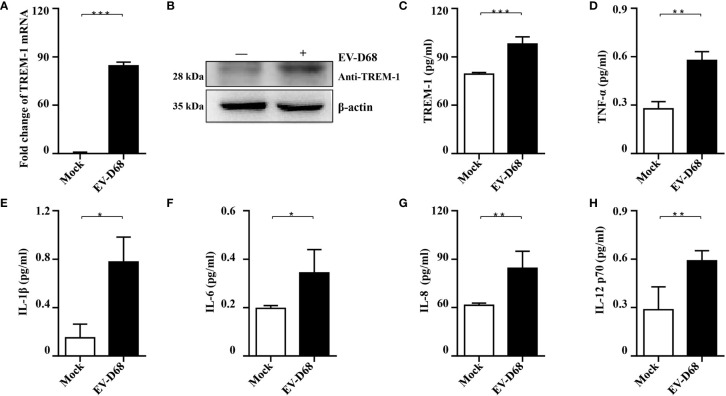
Validation of *RNA-seq data.* ***(*A-C*)*** EV-D68 infection upregulates TREM-1 mRNA and protein abundance in RD cells. EV-D68-infected RD cells were collected at 24 hpi (MOI = 0.1), and TREM-1 mRNA levels were measured by RT-PCR **(A)**. The GAPDH gene served as an internal control, and relative gene expression (fold change) levels of each gene were calculated using the comparative 2^-ΔΔCT^ method. Values are mean ± standard deviation (SD). TREM-1 protein levels were measured by WB **(B)** and ELISA **(C)**. **(D-H)** EV-D68 infection upregulates TREM-1 downstream inflammatory cytokine production. MILLIPLEX assays were used to measure the expression levels of cytokines TNF-α **(D)**, IL-1β **(E)**, IL-6 **(F)**, IL-8 **(G)**, and IL-12 p70 **(H)**. *p <0.05, **p <0.01, ***p <0.001.

To further investigate whether EV-D68 infection influences TREM-1 expression and confirm the accuracy and reliability of the transcriptome data, we infected RD cells with EV-D68 and examined the changes in the RNA and protein levels of TREM-1. Our RT-PCR results showed that TREM-1 mRNA levels were significantly induced upon EV-D68 infection (**Figure 3A**). Consistently, western blotting (WB) and enzyme-linked immunosorbent assay (ELISA) showed that TREM-1 protein levels were elevated by EV-D68 (**Figures 3B, C**). Considering enterovirus caused human respiratory illness, we also evaluated TREM-1 upregulation induced by EV-D68 in human fetal lung diploid fibroblasts (2BS) and human lung carcinoma (A549) cells. RT-PCR results showed EV-D68 infection upregulated TREM-1 mRNA level in 2BS and A549 cells, which proved that is a common phenomenon (**Supplementary Figure 2**). These results suggest that EV-D68 infection upregulated TREM-1 production. Also, we performed ELISA experiments to characterize the change in levels of TREM-1 downstream cytokines, and the results proved that IL-6, IL-8, TNF-α, and others were indeed upregulated in response to viral infection (**Figures 3D–H**).

### Inhibition of TREM-1 Abrogates EV-D68-Mediated Inflammatory Responses

**Figure 4:**
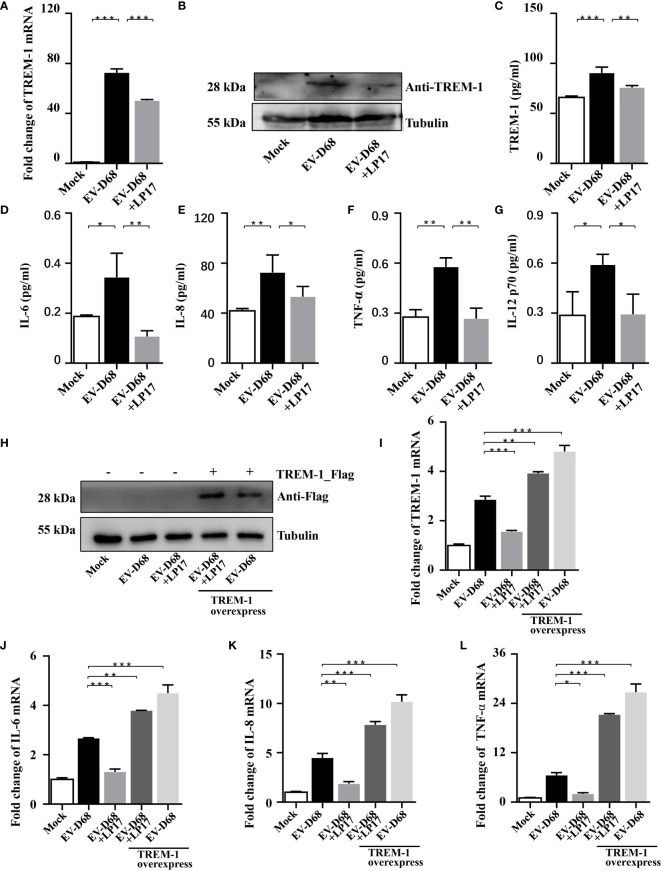
Inhibition of TREM-1 abrogates EV-D68-mediated inflammatory responses. **(A−C)** LP17-mediated down-regulation of TREM-1 in EV-D68-infected RD cells. EV-D68-infected RD cells were pretreated with the TREM-1 inhibitor LP17 for 2 h (1 μg/ml). After 24 h, cells were harvested for subsequent experiments. TREM-1 mRNA levels were measured by RT-PCR (A). The GAPDH gene served as an internal control. The protein levels were measured by WB and ELISA **(B, C)**. **(D−G)** LP17 inhibits the expression of inflammatory cytokines downstream of TREM-1. RD cells in 6-well plates were infected with EVD68 (MOI = 0.1) ± LP17 (1 μg/ml). Cells were collected at 24 hpi for *MILLIPLEX Multiplex Assay* to quantitate changes in the levels of cytokines IL-6 **(D)**, IL-8 **(E)**, TNF-α **(F)**, and IL-12 p70 **(G)**. **(H−L)** TREM-1 over-expression upregulates inflammatory cytokine levels in virus-infected HEK293T cells. HEK293T cells were transfected with TREM-1_Flag or empty vector control (500 ng, 48-well plates) for 24 h. Cells were then infected with EV-D68 (MOI = 0.1) for 24 h, harvested for RNA extraction, and subjected to WB **(H)** and RT-PCR analysis of TREM-1 mRNA **(I)**, IL-6 **(J)**, IL-8 **(K)**, TNF-α **(L)**. The GAPDH gene served as an internal control, and the relative gene expression (fold change) levels of each gene were using with the comparative 2^-ΔΔCT^ method. Values are means ± SD. *p <0.05, **p <0.01, ***p <0.001.

Studies have proved that TREM-1 is a 30 kDa transmembrane immunoreceptor, extensively involved in innate immune and inflammatory responses (22, 23). Furthermore, activation of TREM-1 signaling amplifies proinflammatory responses induced by Toll-like receptors or NOD-like receptor ligands (21, 24). To investigate the molecular basis of TREM-1 regulation of downstream cytokines, we down-regulated endogenous TREM-1 activity using LP17 (25, 26), a molecule targeting TREM-1. We confirmed downregulation of TREM-1 by RT-PCR, WB and ELISA (**Figures 4A–C**). When investigating whether TREM-1 signaling modulates EV-D68-induced inflammatory responses, we found that EV-D68 stimulation of IL-6, IL-8, TNF-α, and IL-12 p70 was abrogated following down-regulation of TREM-1 (**Figures 4D–G**). Additionally, we overexpressed TREM-1 in HEK293T cells, and verified changes in protein abundance by WB (**Figure 4H**). Similarly, we observed that EV-D68 infection led to the upregulation of TREM-1 in HEK293T cells (**Figure 4I**, lane2). Notably, our RT-PCR results demonstrated that overexpression of TREM-1 increased IL-6, IL-8, and TNF-α at the mRNA level (**Figures 4J–L**, lane5), while this amplification effect could be eliminated by LP17 (**Figures 4J–L**, lane4). Collectively, our results suggest that TREM-1 is involved in regulating EV-D68-induced inflammatory responses.

### TREM-1 Inhibition Impacts EV-D68 Replication

**Figure 5:**
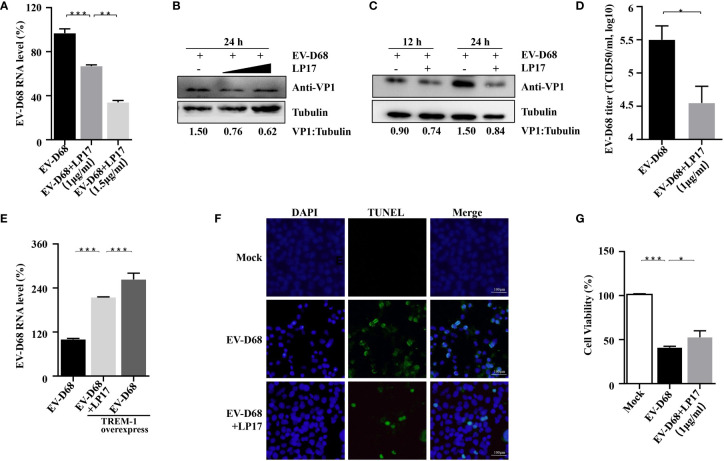
TREM-1 inhibition impacts EV-D68 viral replication. **(A, B)** LP17 inhibits the replication of EV-D68 in a dose-dependent manner. LP17-pretreated RD cells (1 μg/ml or 1.5 μg/ml, 2 h) were exposed to EV-D68 for 24 h Cells were then harvested for RT-PCR analysis of relative EV-D68 RNA levels **(A)**, WB analysis of viral structural protein VP1 **(B)**, and TCID50 analysis of EV-D68 titers **(D)**. **(C)** RD cells were pretreated with LP17 for 2 h (1 μg/ml), then exposed to EV-D68 for 12 h or 24 h Cells were harvested for WB analysis. Tubulin was used as the sample loading control, and VP1 expression levels were normalized and against β-tubulin by ImageJ. **(E)** TREM-1 over-expression upregulates EV-D68 RNA levels. HEK293T cells were transfected with TREM-1_Flag or empty vector control (500 ng, 48-well plates) for 24 h Cells were then infected with EV-D68 (MOI = 0.1) for 24 h, harvested for RNA extraction, and subjected to RT-PCR analysis of relative EV-D68 RNA levels. **(F, G)** Effect of LP17 on apoptosis and cell viability of EV-D68-infected cells. RD cells were infected with EV-D68 following pretreatment with the synthetic peptide LP17 (1.0 μg/ml) for 2 h, and apoptosis and cell viability were determined by TUNEL and CCK-8 assay, respectively. **(F)** Fluorescent signals from TUNEL (green) and DAPI (blue) assays. Bars = 100 μm. The merged image shows the co-localization of nuclei and apoptotic cells. *p <0.05, **p <0.01, ***p <0.001.

Amrun et al. reported that severe EV-A71 infection leads to activation of the TREM-1 signaling pathway, and LP17-mediated TREM-1 inhibition does not affect the replication of EV-A71 (27). Herein, we investigated whether the LP17-mediated down-regulation of the TREM-1 signal pathway affects the replication of EV-D68. First, RD cells were infected with EV-D68 at an MOI of 0.1 after LP17 pretreatment for 2 h. Next, the abundance of viral RNA, the induction of viral protein, and the viral titer of EV-D68 in uninfected control, EV-D68-infected, and LP17 treatment groups were compared. Our RT-PCR results showed that the abundance of EV-D68 RNA in the LP17 treatment group was significantly lower than in the EV-D68-infected group (**Figure 5A**) in a dose-dependent manner. Similarly, WB analysis showed that LP17 significantly inhibited the production of the viral structural protein VP1 (**Figure 5B**, lanes 2 and 3). Furthermore, WB analysis showed that LP17 significantly inhibited the expression of VP1 at 12 hpi and 24 hpi, and the inhibition rate at 24 hpi was ~50%, consistent with the results in **Figure 5B** (**Figure 5C**). Furthermore, as expected, the LP17 treatment group had a markedly lower viral titer than the EV-D68 infected group, and the inhibitory effect was ~90% (**Figure 5D**). To further confirm the correlation between TREM-1 and EV-D68 replication, TREM-1 was overexpressed in HEK293T cells, which were then infected with EV-D68, and the abundance of EV-D68 viral RNA was measured. The results showed that the abundance of EV-D68 RNA was increased significantly following TREM-1 over-expression, and could be inhibited by LP17 (**Figure 5E**). Additionally, the results of Cell Counting Kit 8 (CCK8) and transferase dUTP nick end labeling (TUNEL) experiments showed that the LP17 treatment group displayed a lower rate of apoptosis and higher cell viability, compared with the EV-D68-infected group (**Figures 5F, G**). Taken together, these results suggested that TREM-1 potentially participates in and affects the replication of EV-D68 in host cells.

### Induction of TREM-1 in Response to EV-D68 Infection Is Dependent on NF-κB p65

**Figure 6:**
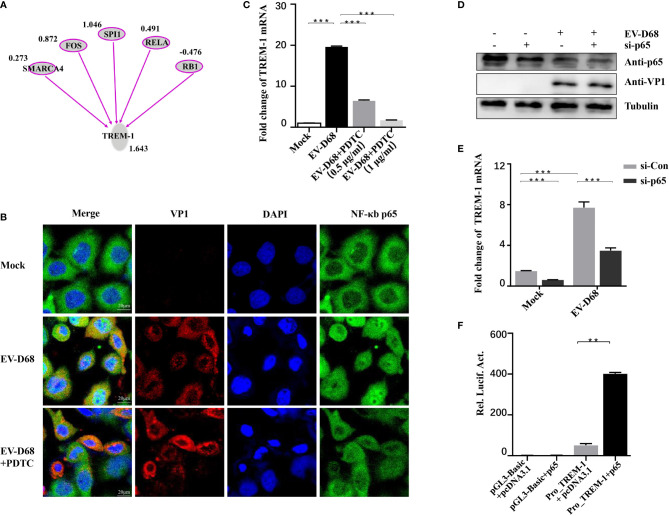
TREM-1 induction by EV-D68 is dependent on NF-κB p65. **(A)** Analysis of TREM-1 upstream regulators using IPA. **(B, C)** Hela cells were pre-incubated with PDTC (500 ng/ml, 48-well plates) for 2 h and EV-D68 was added (MOI = 0.1) for 24 h. A laser confocal microscope was used to observe p65 nuclear import and RT-PCR analysis was performed to measure TREM-1 mRNA abundance. The GAPDH gene served as an internal control. **(D, E)** HEK-293T cells were transfected with si-p65 (100 nM) for 24 h, and then infected with EV-D68 (MOI = 0.1). SDS-PAGE analysis was conducted to measure NF-κB p65 expression levels **(D)** and qPCR was performed to measure TREM-1 mRNA levels **(E)**. The GAPDH gene served as an internal control. **(F)** NF-κB p65 or pcDNA3.1 was cotransfected with Pro_TREM-1 or pGL3-Basic in HEK293T cells (24-well plate). Empty vectors pGL3-Basic and pcDNA3.1 were used as controls, and all cells were transfected with pRL-SV40 as an intracellular reference. After 48 h, a dual-reporter detection system was used to measure luciferase expression levels of different groups. **p <0.01, ***p <0.001.

IPA analysis revealed that TREM-1 upstream regulators were significantly activated in response to EV-D68 infection, including SWI/SNF-related, matrix-associated, actin-dependent regulator of chromatin, subfamily a, member 4 (SMARCA4), FOS, SPI1, NF-κB p65, and RB transcriptional corepressor 1 (RB1) (**Figure 6A**). Zeng et al. reported that NF-κB p65 regulates the expression of TREM-1 in mouse-derived cells (28), but the promoter sequence similarity among different species is low based on Basic Local Alignment Search Tool (BLAST) searches. Therefore, we explored whether NF-κB p65 is involved in regulating the expression of TREM-1. We first confirmed that nuclear translocation of NF-κB p65 was induced by EV-D68 (**Figure 6B**, row 2). Activation of NF-κB was alleviated by pyrrolidine dithiocarbamate (PDTC), a selective NF-κB p65 inhibitor, resulting in a decrease in TREM-1 mRNA level (**Figure 6C**, lanes 3−4). To further define the regulation between TREM-1 and NF-κB p65, we silenced the p65 gene using a small interfering RNA (siRNA) approach (**Figure 6D**). NF-κB p65 knockdown cells were infected with EV-D68, and expression of the TREM-1 gene was measured. We observed that p65 silencing resulted in an approximately 50% decrease in TREM-1 mRNA levels (**Figure 6E**, lane 4). Next, we performed a dual-luciferase assay to determine whether NF-κB p65 could bind to the promoter region of the endogenous TREM-1 gene. **Figure 6F** shows that TREM-1 expression was regulated at a transcriptional level by NF-κB p65. Together, these results indicate that activation of NF-κB p65 is essential for expressing the TREM-1 gene in response to EV-D 68 infection.

### Activated TREM1 Signals Through the p38 MAPK Pathway

**Figure 7:**
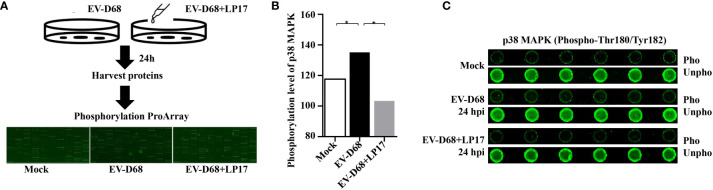
Activated TREM-1 signals through the p38 MAPK pathway. **(A)** Flow chart of Phosphorylation ProArray (CSP100_plus) detection. The experiment included six replicates (horizontal rows) of phospho-specific and total antibodies, which were assessed against different treatment groups. **(B, C)** The graph shows the levels of TREM-1 downstream p38 MAPK phosphorylation level for Mock, EV-D68, and EV-D68+LP17 groups. *p <0.05.

**Figure 8:**
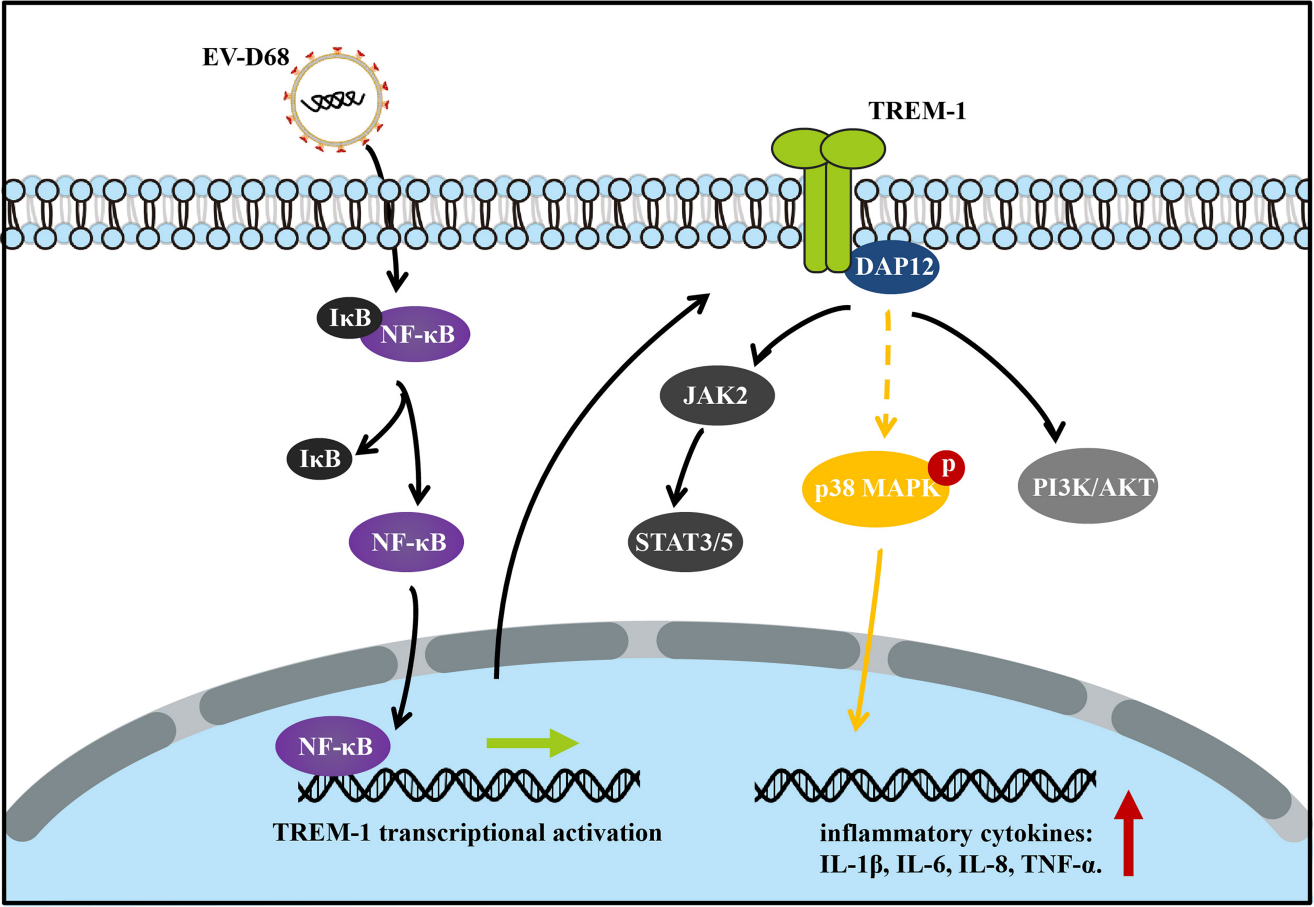
Schematic diagram of the activated TREM-1 signal pathway during EV-D68 infection. Transcriptional activation of TREM-1 is induced by EV-D68, and TREM-1 then regulates the secretion of downstream cytokines including IL-6, IL-8, and TNF-α.

After ligand binding, activation of TREM1 signaling is mediated by homotypic interactions between the immunoreceptor tyrosine-based activation motifs (ITAM) between TREM1 and the adaptor DNAX-Activation Protein 12 (DAP12) (29). This interaction results in phosphorylation of DAP12, which facilitates the recruitment of Src family kinases to activate downstream signaling involving Janus kinase (JAK), signal transducers and activators of transcription 3/5 (STAT3/5), MAPK, and phosphatidylinositol 3 kinase/AKT (PI3K/AKT) (29–32). Next, we identified TREM-1 downstream targets by directly comparing the phosphorylation levels of the above candidate signals between groups (**Figure 7A**). Using large-scale proteomic screen antibody arrays, we discovered that EV-D68 significantly increased phosphorylation of p38 MAPK (phospho-Thr180/Tyr182), which implied that TREM-1 transmits activation signals to phosphorylate p38 MAPK (**Figure 7B**, lane 2 and **Supplementary Table 2**). The involvement of p38 MAPK was further confirmed through studies utilizing LP17. Phosphorylation levels of p38 MAPK (phospho-Thr180/Tyr182) were decreased ~0.76-fold in the LP17-treated group compared with the EV-D68 infection group (**Figure 7B**, lane 3). **Figure 7C** shows the phosphorylation modification and significant changes in p38 MAPK. This result indicates that EV-D68 infection activates TREM-1 and leads to phosphorylation of downstream p38 MAPK, which is potentially involved in the regulation of downstream cytokines (33). Taken together, our study demonstrated that EV-D68 infection stimulated NF-κB p65 nuclear import to activate TREM-1 transcription, and then TREM-1 transmitted activation signals to phosphorylate p38 MAPK to involve host inflammatory responses. Based on these results, we developed a schematic diagram of the activated TREM-1 signal pathway during EV-D68 infection (**Figure 8**).

## Discussion

In recent years, EV-D68 has advanced from a rarely detected respiratory virus to a widespread pathogen responsible for increasing rates of severe respiratory illness and AFM in children worldwide, making it a serious threat to public health. To date, no specific antiviral medicines or treatments have been approved for EV-D68 infection, partly due to the lack of in-depth understanding of the pathogenic mechanism of the virus. In the present study, we generated a transcriptomic landscape map of EV-D68-infected RD cells to reveal the molecular mechanisms responsible for viral infections. We applied IPA to construct a GRN, analyzed the activation of genes and their upstream regulatory factors, and further verified our hypotheses through *in vitro* experiments. In summary, we found that TREM-1, TNF, and NF-**κ**B are the major regulators in the gene regulation network connecting proinflammatory responses. Thus, TREM-1 signaling pathway is an important mediator of inflammatory responses associated with EV-D68 infections.

Transcript profiling showed that EV-D68 infection significantly stimulated host immune responses, resulting in an activated antiviral state in infected cells. EV-D68 infection triggered activation of transmembrane receptors (TREM-1, TLR3/4/7, IL-1R), transcription factors (RELA, STAT3, JunB), and cytokines (TNF, IL, CCL5). IPA-based analysis showed that these factors interact through a GRN, and are involved in the host inflammatory response. Recently developed animal models of EV-D68 infection have provided limited information about the pathogenesis of EV-D68-induced airway disease. Nasal inoculation of cotton rats with a recently isolated EV-D68 strain (VANBT/1) significantly upregulated pulmonary cytokine mRNA levels (CCL2, CCL5, CXCL1, CXCL10, IL-6, IFN-β, and IFN-γ) and provided histological evidence of peribronchiolitis and alveolitis (34). Nasal infection of ferrets with the EV-D68 Fermon strain increased the abundance of lung proteins IL-1α, IL-8, and IL-12 p70 during early infection, and lung pathology was continuously exacerbated during the progression of infection, causing lung edema as well as lung injury at the middle and late stages of infection (35). These reports explored the abdominal inflammatory pathological response induced by EV-D68 infection, and suggest that inflammation is involved in pathophysiological processes caused by EV-D68 infection. Rajput et al. found that treatment with anti-IL-17 antibody reduced the expression of lung cytokines (TNF-α, IL-12b, and IL-17A) and chemokines (CCL2, CXCL1, CXCL2, and CXCL10), and attenuated EV-D68-induced airway responsiveness in neutrophilia (36). However, the specific mechanism needs to be further investigated.

Similar to PRRs, activation of TREM-1 signaling is initiated upon binding of the ligand to the receptor, which triggers the association and phosphorylation of the immunoreceptor tyrosine-based activation motif of the adaptor protein DAP12, resulting in the recruitment and activation of the non-receptor tyrosine kinase Syk (29). In turn, Syk activates downstream signaling molecules including PI3K, PLCγ, ERK1/2, and MAP kinases (37, 38).

Regarding the role of TREM-1 in the inflammatory response, it may be involved in the pathophysiology of the inflammatory cascade caused by EV-D68 infection, and the results of LP17 targeting of TREM-1 in the present work support this view. We used the selective inhibitor LP17 to down-regulate TREM-1, and downstream cytokine secretion was consequently decreased, and TREM-1 over-expression promoted the transcriptional activity of downstream cytokines. These results implicate TREM-1 in the regulation of the inflammatory response caused by EV-D68 infection.

Various viruses have been shown to increase the expression of TREM-1 mRNA and soluble TREM-1, including filoviruses (Marburg and Ebola) (39) and flaviviruses (human Dengue virus and West Nile Virus) (40, 41). However, it is not yet established whether activation of TREM-1 is pathogenic or protective in viral infections. Weber et al. recently showed that TREM-1 knockout protects against severe influenza infection, although viral clearance was unaltered in knockout animals (42). These findings imply that limiting inflammation by blocking TREM-1 in the setting of acute viral infection may contribute to its protective effect. However, our current study found that LP17 inhibited EV-D68 replication in RD cells, at both virus RNA and protein/particle levels. This phenomenon provides a molecular basis for TREM-1 as a combined target for antiviral therapy.

Overall, our study is the first to show that TREM-1 is induced by EV-D68. We found that TREM-1 was involved in inflammatory responses and regulated the secretion of downstream cytokines including IL-6, IL-8, TNF-α, and others. Additionally, NF-κB p65 was essential for EV-D68-induced TREM-1 up-regulation based on NF-κB p65 activation inhibitor and small interfering RNA (siRNA) treatments. A dual-luciferase assay demonstrated that NF-κB p65 binds to the promoter region of the endogenous TREM-1 gene and regulates TREM-1 expression at the transcriptional level. Furthermore, inhibition of the TREM1 signaling pathway with the specific inhibitor LP17 dampened activation of the p38 MAPK signaling cascade, which suggests that TREM-1 mainly transmits activation signals to phosphorylate p38 MAPK. Increasingly, studies are exploring the role of inflammatory responses in antiviral immunity. For example, Rao et al. applied a cell bait approach to deceive coronavirus, reduce inflammatory cytokine production, and eliminate lung damage caused by SARS-Cov-2 infection (43). However, a deeper conceptual understanding of the mechanisms associated with the pathogenic and protective functions of TREM-1 in antiviral immunity is essential to develop novel therapeutic strategies for controlling virus infection by modulating innate immune signaling.

## Data Availability Statement

The datasets presented in this study can be found in online repositories. The names of the repository/repositories and accession number(s) can be found below: Baidu Drive with a permanent sharing link: https://pan.baidu.com/s/1HbjQLyxU3lEsp2C7PL_3Vw (extraction code: 2021).

## Author Contributions

YT, TW and YJS contributed to the design of the study; YT, SY, JYL, RCL, YJC and SHL contributed to data analysis; TW, SHL, RCL, YLC, HYL, and YZS contributed to data interpretation; YT, SY, JYL and TW contributed to writing the manuscript. All authors contributed to the article and approved the submitted version.

## Funding

This work was supported by the National Key Research and Development Program of China (2017YFA0205102), the State Key Laboratory of Pathogen and Biosecurity Program (SKLPBS2110), and the National Science and Technology Major Project (2018ZX10101003-002-011).

## Conflict of Interest

The authors declare that the research was conducted in the absence of any commercial or financial relationships that could be construed as a potential conflict of interest.

## Publisher’s Note

All claims expressed in this article are solely those of the authors and do not necessarily represent those of their affiliated organizations, or those of the publisher, the editors and the reviewers. Any product that may be evaluated in this article, or claim that may be made by its manufacturer, is not guaranteed or endorsed by the publisher.

## Supplementary Material

Supplementary Figure 1 GO enrichment analysis of upregulated genes in mock vs. 6 hpi **(A)**, mock vs. 12 hpi **(B)**, and mock vs. 24 hpi [**(C)** groups]. The dot diameter represents the number of DEGs; color depth represents significance; the abscissa represents the ratio of the number of DEGs annotated to GO terms to total DEGs; the ordinate represents different GO terms.

Supplementary Figure 2 EV-D68 infection upregulates TREM-1 mRNA abundance in A549 and 2BS cells. **(A, B)** EV-D68-infected A549 and 2BS cells were collected at 24 hpi (MOI = 0.05, 0.1, 0.5, 1), and TREM-1 mRNA levels were measured by RT-PCR. The GAPDH gene served as an internal control, and relative gene expression (fold change) levels of each gene were calculated using the comparative 2^-ΔΔCT^ method. Values are means ± SD. **p <*0.05, ***p <*0.01, ****p <*0.001.

Supplementary Table 1 Primer sequences used for RT-PCR.

Supplementary Table 2 Phosphorylation levels of the candidate signals downstream of TREM-1.

Supplementary Data Sheet 1 Gene expression levels of mock and EV-D68-infected RD cells at 6 hpi, 12 hpi, 24 hpi.

Supplementary Data Sheet 2 DEGs list at 6 hpi.

Supplementary Data Sheet 3 DEGs list at 12 hpi.

Supplementary Data Sheet 4 DEGs list at 24 hpi.
