# Supplementary figures and images for: Transcriptomic Profiling Reveals a Role for TREM-1 Activation in Enterovirus D68 Infection-Induced Proinflammatory Responses
Source: Front Immunol. 2021 Nov 23;12:749618. doi: 10.3389/fimmu.2021.749618 (PMC8650217; doi:10.3389/fimmu.2021.749618)

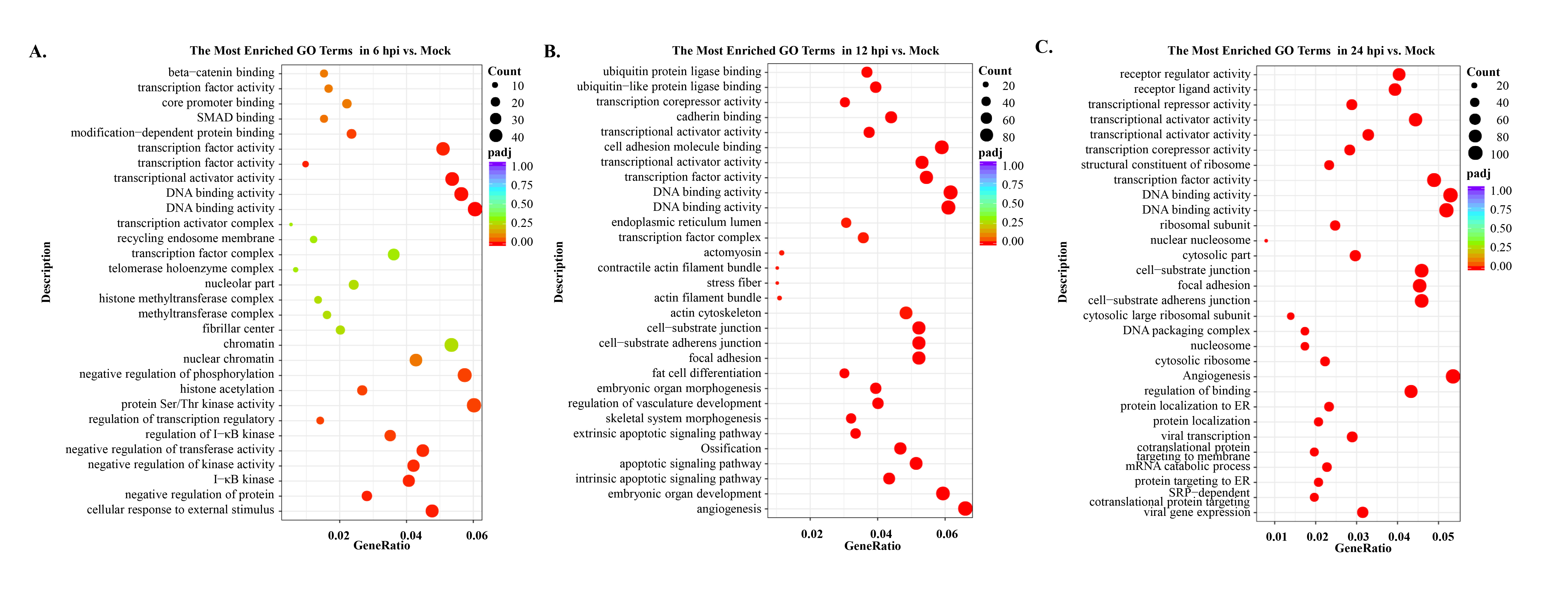

Supplement: Supplementary Figure 1 — GO enrichment analysis of upregulated genes in mock vs. 6 hpi (A), mock vs. 12 hpi (B), and mock vs. 24 hpi [(C) groups]. The dot diameter represents the number of DEGs; color depth represents significance; the abscissa represents the ratio of the number of DEGs annotated to GO terms to total DEGs; the ordinate represents different GO terms. [file Image_1.jpg]

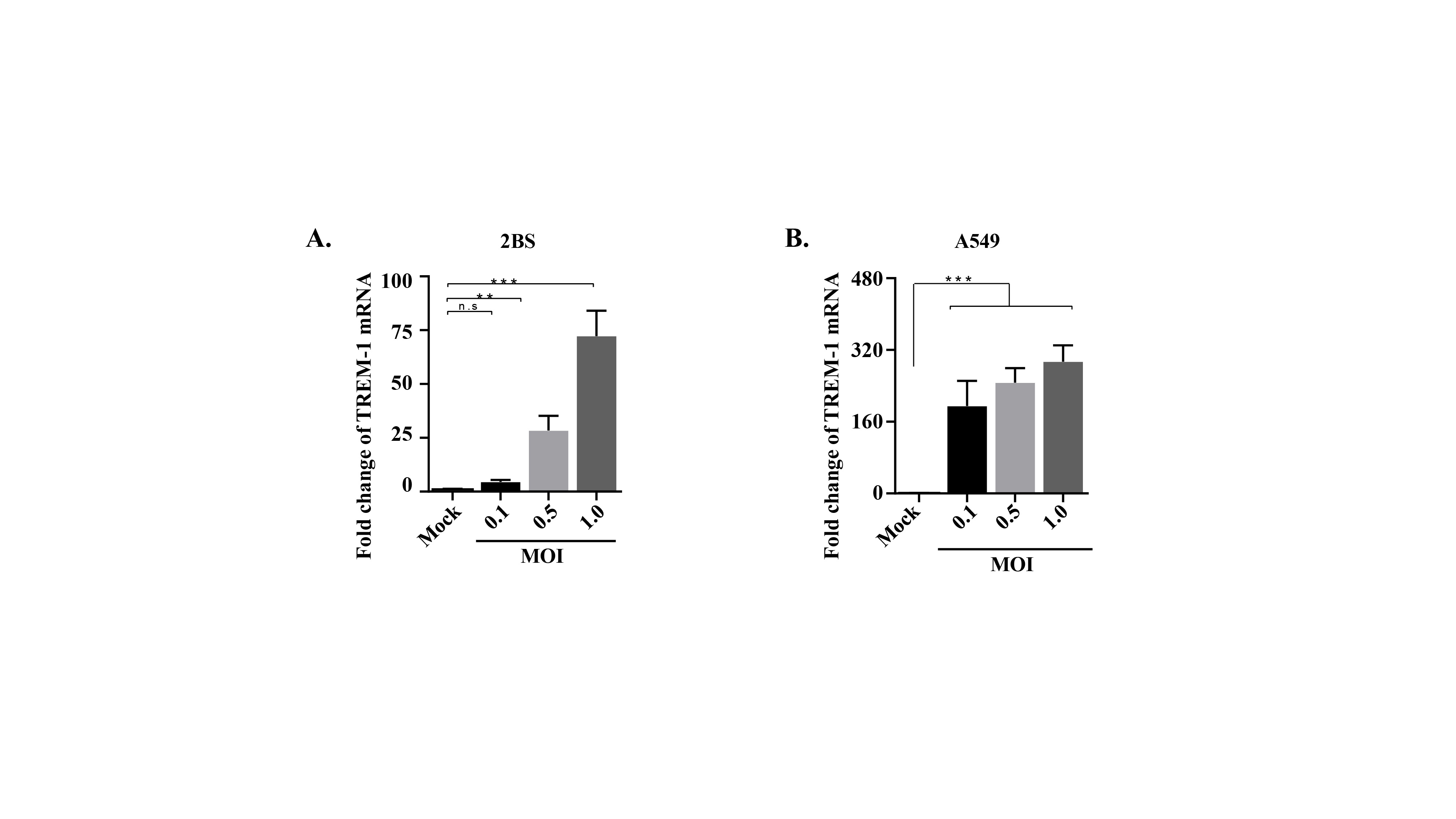

Supplement: Supplementary Figure 2 — EV-D68 infection upregulates TREM-1 mRNA abundance in A549 and 2BS cells. (A, B) EV-D68-infected A549 and 2BS cells were collected at 24 hpi (MOI = 0.05, 0.1, 0.5, 1), and TREM-1 mRNA levels were measured by RT-PCR. The GAPDH gene served as an internal control, and relative gene expression (fold change) levels of each gene were calculated using the comparative 2-ΔΔCT method. Values are means ± SD. *p <0.05, **p <0.01, ***p <0.001. [file Image_2.jpg]
