# Supplementary material for: Transcriptomic Profiling Reveals a Role for TREM-1 Activation in Enterovirus D68 Infection-Induced Proinflammatory Responses
Source: Front Immunol. 2021 Nov 23;12:749618. doi: 10.3389/fimmu.2021.749618 (PMC8650217; doi:10.3389/fimmu.2021.749618)
Supplement: Supplementary Table 1 — Primer sequences used for RT-PCR. [file Table_1.docx]

**Supplementary Table 1**. Primer sequences used for RT-PCR.

| genes | primer sequences |
| --- | --- |
| EV-D68 RNA | Forward:5’-GGCAGCCTATCAGGTGGAGAG-3’;  Reverse:5’-GAGTTTGTATGGCTTCTTCTGGT-3’. |
| TREM-1 | Forward: 5’-TGGTCTTCTCTGTCCTGTTTG-3’;  Reverse: 5’-ACTCCCTGCCTTTTACCTC-3’. |
| GAPDH | Forward: 5’-AATCCCATCACCATCTTC-3’;  Reverse: 5’-AGGCTGTTGTCATACTTC-3’. |
| IL-6 | Forward;5’-AAGCCAGAGCTGTGCAGATGAGTA-3’;  Reverse; 5’-TGTCCTGCAGCCACTGGTTC-3’. |
| NF-κB p65 | Forward: 5’-AGCTCAAGATCTGCCGAGTG-3’;  Reverse: 5’-ACATCAGCTTGCGAAAAGGA-3’. |
| IL-8 | Forward: 5’-CTGATTTCTGCAGCTCTGTG-3’;  Reverse: 5’-TTCACTGGCATCTTCACTG-3’. |
| TNF-α | Forward: 5’-AGCCCATGTTGTAGCAAACC-3’;  Reverse: 5’-TGAGGTACAGGCCCTCTGAT-3’. |
| IL-12 p35 | Forward: 5’-TGGCAGTTATTGATGAGC-3’;  Reverse: 5’-TTAGGAAGCATTCAGATAGC-3’. |
| IL-12 p40 | Forward: 5’-CAGAGCAGTGAGGTCTTAGGC-3’;  Reverse: 5’-AAGCAGCAGGAGCGAATGG-3’. |
