# Supplementary material for: Transcriptomic Profiling Reveals a Role for TREM-1 Activation in Enterovirus D68 Infection-Induced Proinflammatory Responses
Source: Front Immunol. 2021 Nov 23;12:749618. doi: 10.3389/fimmu.2021.749618 (PMC8650217; doi:10.3389/fimmu.2021.749618)
Supplement: Supplementary Table 2 — Phosphorylation levels of the candidate signals downstream of TREM-1. [file Table_2.docx]

**Supplementary Table 2**. Phosphorylation levels of the candidate signals downstream of TREM-1.

|  | **Phosphorylation levels** | | | **Phosphorylation ratio** | |
| --- | --- | --- | --- | --- | --- |
|  | **mock** | **EV-D68** | **EV-D68+LP17** | **EV-D68/mock** | **EV-D68+LP17/EV-D68** |
| JAK2 (Phospho-Tyr1007) | 79.86 | 64.61 | 69.43 | 0.81 | 1.07 |
| JAK2 (Phospho-Tyr221) | 80.48 | 56.05 | 63.38 | 0.70 | 1.13 |
| LYN (Phospho-Tyr507) | 81.71 | 78.33 | 77.45 | 0.96 | 0.99 |
| p38 MAPK (Phospho-Thr180/Tyr182) | 117.64 | 135.28 | 103.47 | 1.15 | 0.76 |
| p44/42 MAP Kinase (Phospho-Tyr204) | 69.60 | 55.56 | 60.47 | 0.80 | 1.09 |
| STAT3 (Phospho-Tyr705) | 98.05 | 60.86 | 80.44 | 0.62 | 1.32 |
| STAT5A (Phospho-Tyr694) | 72.06 | 52.98 | 63.08 | 0.74 | 1.19 |
| STAT5B (Phospho-Ser731) | 65.08 | 56.21 | 57.48 | 0.86 | 1.02 |
| AKT1 (Phospho-Ser473) | 68.37 | 57.95 | 57.33 | 0.85 | 0.99 |
| AKT1 (Phospho-Thr308) | 78.84 | 72.20 | 63.83 | 0.92 | 0.88 |
| AKT2 (Phospho-Ser474) | 67.50 | 55.56 | 64.50 | 0.82 | 1.16 |
